# Patient activation in adults attending appointments in general practice: a cross-sectional study

**DOI:** 10.1186/s12875-023-02102-9

**Published:** 2023-07-10

**Authors:** Ingvild Hernar, Marit Graue, Jannicke Igland, David A. Richards, Hilde Kristin Refvik Riise, Anne Haugstvedt, Beate-Christin Hope Kolltveit

**Affiliations:** 1 Department of Health and Caring Sciences, Western Norway University of Applied Sciences, P.O. Box 7030, N-5020 Bergen, Norway; 2 Department of Internal Medicine, Haukeland University Hospital, Bergen, Norway; 3 Department of Global Public Health and Primary Care, University of Bergen, Bergen, Norway; 4 Institute for Health Research, College of Medicine and Health,, University of Exeter, Exeter, UK; 5 Vossevangen Medical Centre, Voss, Norway

**Keywords:** Patient activation, Adults, General practice, Health-related behaviours, Quality of life, Satisfaction with health, Type 2 diabetes, Risk of diabetes

## Abstract

**Background:**

Patient activation refers to patients’ knowledge, confidence, skills, ability, beliefs, and willingness to manage their health and healthcare. Patient activation is an essential component of self-management and identifying patient activation levels will identify people at risk for health decline at an earlier stage. We aimed to explore patient activation in among adults attending general practice by (1) investigating differences in patient activation according to characteristics and markers of health-related behaviour; (2) examining the associations of quality of life and satisfaction with health with patient activation; and (3) comparing patient activation between persons with or without type 2 diabetes (T2D) and with or without elevated T2D risk.

**Methods:**

We performed a cross-sectional study and recruited 1,173 adult patients from four Norwegian general practices between May to December 2019. The participants completed a questionnaire containing sociodemographic and clinical variables, the Patient Activation Measure (PAM-13), the quality of life and satisfaction with health items from the WHO Quality of Life-BREF, three questions about exercise (regularity, intensity and exercise time), the Finnish Diabetes Risk Score (FINDRISC) and Body Mass Index. We tested differences between groups and associations using Chi-squared tests, Fisher’s exact tests, t-tests, one-way ANOVAs and Spearman’s rho tests.

**Results:**

The sample’s mean PAM-13 score was 69.8 (0–100) (SD 14.8). In the total population, we found that participants reporting higher patient activation scores also reported more favourable health-related behaviours (exercise and healthy eating). We found positive correlations between the PAM-13 scores and, respectively, the quality of life score and the satisfaction with health score. We found no differences in patient activation between people with or without T2D and those with or without elevated T2D risk.

**Conclusions:**

We found that higher patient activation was associated with favourable health-related behaviours, a better quality of life and better satisfaction with health among adults attending four general practices in Norway. Assessing patient activation has the potential to help general practitioners identify patients who might benefit from closer follow-up in advance of negative health outcomes.

**Supplementary Information:**

The online version contains supplementary material available at 10.1186/s12875-023-02102-9.

## Introduction

Patient activation refers to patients’ knowledge, confidence, skills, ability, beliefs and willingness to manage their health and healthcare [1, 2]. The concept is considered latent and behavioural, assessing how individuals self-manage their health and how they understand their role and engage as active partners in their health care. Patient activation is essential to self-management, especially for people with health-related challenges and chronic diseases who must attain sufficient knowledge about their condition and treatment to, conceivably, change unfavourable health-related behaviours and integrate new routines in everyday life [3].

According to previous research, chronically ill people reporting lower patient activation levels are more likely to have been hospitalised or had an emergency department visit than those reporting higher levels [4–7]. They are also more likely to develop other chronic diseases [6]. A longitudinal study among people with chronic conditions has indicated that when patient activation changes, health outcomes change in the same direction [8]. Also, increased depressive symptoms and poorer quality of life have been associated with lower patient activation among patients in general practice [9]. Further, patient activation is found to be positively associated with better self-reported health [10, 11]. In addition to identifying patient activation levels to detect people at risk for health decline at an earlier stage, patient activation is a reliable driver of decision-making and improved clinical outcomes [12].

Diabetes, prediabetes and obesity are conditions where patient activation is central for self-management and the individuals’ ability to keep up with health-promoting behaviours to self-manage the condition [12]. Diabetes is estimated to affect 536 million adults worldwide, of which about 90% have type 2 diabetes (T2D) [13]. The risk of developing T2D increases dramatically among people with obesity (Body Mass Index [BMI] ≥ 30 kg/m^2^) [14]. Also, unfavourable lifestyles such as unhealthy diet, lack of regular physical activity and smoking are markers of health-related behaviours associated with poorer health outcomes and increased risk of T2D [15].

Diabetes treatment requires the person to make choices and take actions related to their lifestyle and medication management [16]. Poor diabetes management can lead to significant morbidity with increased hospitalisation rates, greater personal and societal costs and increased mortality [17, 18]. Recent findings suggest that the relationship between self-management and patient activation is mediated by self-efficacy [19]. Adding motivational and psychological factors to personalised care interventions have the potential to improve self-efficacy and provide patients with emotional support and disease knowledge [19]. Among people with T2D, increased patient engagement and activation are found to improve blood pressure, lipids and glycaemic control (Haemoglobin A_1c_) [4, 7, 11, 20]. Therefore, recognising patient activation levels in follow-up in general practice could identify people at risk for health decline at an earlier stage. However, we have limited knowledge about patient activation among Norwegian adults in general and among patients attending general practice.

This study is part of a larger study and was designed to identify eligible participants for a randomised control trial (RCT) aiming to improve patient activation among adults attending general practice by promoting knowledge, skills, and confidence integral to managing one’s health and healthcare and facilitating for self-management and lifestyle change to avoid further health challenges (ClinicalTrials.gov ID: NCT04076384). In this study, we aimed to explore patient activation in a sample of adults attending follow-up in general practice by (1) investigating differences in patient activation according to sociodemographic characteristics, clinical characteristics, and markers of health-related behaviour; (2) examining the associations of quality of life and satisfaction with health with patient activation; and (3) comparing patient activation between persons with or without T2D and between persons with or without elevated T2D risk. We report design and results using the STROBE reporting guidelines for cross-sectional studies [21].

## Methods

### Study design, participants and setting

The study had a cross-sectional design. We recruited the study sample from four general practices in Western and South-Eastern Norway between May and December 2019. According to the sample size calculations for the planned RCT, we needed to recruit at least 1,400 participants to be able to identify enough participants for the intervention study. Potentially eligible participants were consecutively identified by a study nurse and approached in the waiting area. We applied the inclusion and exclusion criteria for the planned RCT. The inclusion criteria were: 1) adults aged 20–80 years, 2) attending a consultation with a general practitioner (GP). We excluded people with serious somatic illnesses (e.g., severe cancer, severe heart failure, end-stage renal disease), major psychiatric disorders (e.g., severe depression, bipolar disorder, schizophrenia), recorded cognitive deficiency (e.g., Down’s syndrome, Alzheimer’s disorder), pregnancy or not being able to read, speak or understand Norwegian.

### Data collection and variables

The study nurse handed out a self-report questionnaire on paper to the patients that consented. We collected the participants’ sociodemographic and clinical characteristics: sex, age, living situation, educational level, work situation, smoking habits and known diabetes (yes/no). We used the generic Patient Activation Measure® (PAM-13) to assess the participant’s knowledge, skills and confidence in managing their health and preventing health problems [22, 23]. The PAM-13 consists of 13 items, e.g., “I am the person who is responsible for taking care of my health”, “I am confident that I can carry out medical treatments I may need to do at home”, “I know how to prevent problems with my health”. The response options range from *Strongly agree* (4) to *Strongly disagree* (1), with the alternative *Not applicable*. Item scores are summed and transformed into a 0–100-point scale where higher scores represent higher patient activation. The total score is divided into four patient activation levels. Patients at level 1 (score 0–47.0) are described as *Disengaged and overwhelmed*; at level 2 (47.1–55.1) *Becoming aware, but still struggling*; at level 3 (55.2–67.0) *Taking action and gaining control* and at level 4 (67.1–100) *Maintaining behaviours and pushing further* [24]. The questionnaire is translated into Norwegian, displaying acceptable psychometric properties, and is deemed suitable for clinical use and research [25, 26].

We also collected the two global items from the WHO Quality of Life-BREF (WHOQOL-BREF) [27]. The participants were asked to rate their quality of life from *very poor to very good* (1–5) and their satisfaction with health from *very dissatisfied to very satisfied* (1–5). The questionnaire is translated into Norwegian and has satisfactory psychometric properties [28]. Further, we included three questions regarding exercise (regularity, intensity and average time spent exercising) from the Trøndelag Health Study (HUNT4) [29]. Finally, the participants completed the Finnish Diabetes Risk Score (FINDRISC), a widely used assessment tool for measuring the respondents’ risk for developing T2D [30, 31]. The FINDRISC covers eight known risk factors: age, BMI, waist circumference, daily physical activity, daily intake of vegetables, fruits and berries, history of antihypertension drug treatment, history of hyperglycaemia and family history of diabetes. The risk score ranges from 0 to 26 (items are weighted differently) [31]. Scores ≥ 15 have been applied for identifying T2D risk [31, 32]. In the present study, the study nurse assisted with measuring weight, height and waist circumference and calculated BMI.

### Statistical analyses

We undertook descriptive analyses (count, proportion, mean and standard deviation [SD]) to quantify sample characteristics and questionnaire scores. Participants completing fewer than seven of the PAM-13 items were excluded from the analyses. Using t-tests, one-way ANOVAs, Chi-squared tests and Fisher’s exact tests, we examined differences in PAM-13 scores according to sociodemographic characteristics, clinical characteristics, and health-related behaviours. Next, we calculated Spearman’s rho to assess the correlations between PAM-13 scores and, respectively, quality of life and satisfaction with health scores. Also, we performed one-way ANOVAs to identify the associations of quality of life and satisfaction with health (WHOQOL-BREF) scores (dependent variables) with the four PAM-13 levels (independent variable). Furthermore, we defined participants with FINDRISC ≥ 15 and/or BMI ≥ 30 kg/m^2^ as having elevated T2D risk. We chose the FINDRISC cut-off in accordance with previous studies [31, 32]. BMI ≥ 30 kg/m^2^ was chosen since previous studies report that T2D risk increases significantly at this level [14, 15]. Finally, we used t-tests and Chi-squared tests to compare PAM-13 scores and levels between participants with and without diabetes and those with and without elevated T2D risk. We used Stata SE 16.0 and MP 17.0 for analyses and defined the significance level as *p* < 0.05.

## Results

**Fig. 1:**
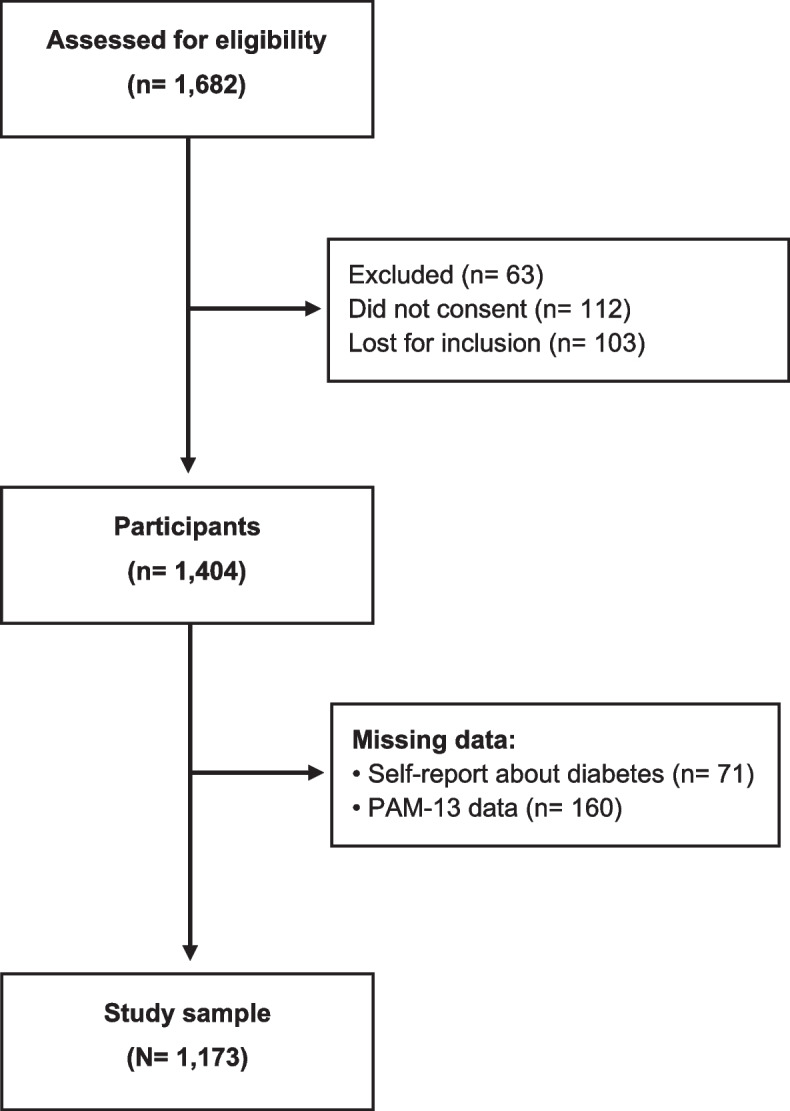
Flow chart of the recruitment and inclusion of the study sample

In total, we identified 1,682 potentially eligible participants, of which 63 were later excluded, 112 declined participation, and 103 were lost because of organizational challenges, leaving 1,404 recruited (83.5% participation rate) (Fig. 1). After excluding 71 participants due to missing diabetes status and 160 with more than seven missing PAM-13 items, the study sample was reduced to 1,173 participants. Sociodemographic and clinical characteristics and markers of health-related behaviours are presented in Table 1. In brief, women constituted 53.6% of the sample (*n* = 629), and the participants’ mean age was 54.9 years (SD 16.0). Furthermore, 253 (21.6%) had BMI ≥ 30 kg/m^2^, and the mean FINDRISC score was 9.5 (SD 5.3).

**Table 1:** The study sample’s sociodemographic characteristics, clinical **characteristics** and health-related behaviours

	**Total study sample**	**Self-reported diabetes**			**Elevated T2D risk**^**a**^		
		**Yes**	**No**		**Yes**	**No**	
	*N* = 1,173	*n* = 128	*n* = 1,045	*p*	*n* = 256	*n* = 789	*p*
Sex				.495			.866
Men	544 (46.4)	63 (49.2)	481 (46.0)		119 (46.5)	362 (45.9)	
Women	629 (53.6)	65 (50.8)	564 (54.0)		137 (53.5)	427 (54.1)	
Age (years)	54.9 ± 16.0	62.7 ± 11.2	53.9 ± 16.3	< .001	55.6 ± 15.5	53.3 ± 16.4	.044
Age categories				< .001			.130
< 45 years	305 (26.9)	10 (7.8)	295 (28.2)		64 (25.0)	231 (29.3)	
45–54 years	212 (18.1)	16 (12.5)	196 (18.8)		53 (20.7)	143 (18.1)	
55–64 years	271 (23.1)	42 (32.8)	229 (21.9)		48 (18.8)	181 (22.9)	
> 64 years	385 (32.8)	60 (46.9)	325 (31.1)		91 (35.5)	234 (29.7)	
Living situation				.172			.034
Live with others	841 (71.7)	87 (68.0)	754 (72.1)		170 (66.4)	584 (74.0)	
Live alone	229 (19.5)	31 (24.2)	198 (19.0)		59 (23.1)	139 (17.6)	
Educational level				.062			< .001
Primary/secondary school	814 (69.4)	98 (76.6)	716 (68.5)		198 (77.3)	518 (65.7)	
University/college education	359 (30.6)	30 (23.4)	329 (31.5)		58 (22.7)	271 (34.3)	
Work situation^b^				.009			.002
Fulltime work	509 (43.4)	43 (33.6)	466 (44.6)		100 (39.0)	366 (46.4)	
Part-time work	138 (11.7)	10 (7.8)	128 (12.2)		26 (10.2)	102 (12.9)	
On benefits	106 (9.0)	17 (13.3)	89 (8.5)		36 (14.1)	53 (6.7)	
Retired	361 (30.8)	53 (41.4)	308 (29.5)		83 (32.4)	225 (28.5)	
Other	56 (4.8)	5 (3.9)	51 (4.9)		10 (3.9)	41 (5.2)	
Smoking habits				.042			.059
Never smoked	436 (37.2)	40 (31.3)	396 (37.9)		88 (34.4)	308 (39.0)	
Former smoker	556 (47.4)	74 (57.8)	482 (46.1)		134 (52.3)	348 (44.1)	
Current smoker	178 (15.1)	14 (10.9)	164 (15.7)		33 (12.9)	131 (16.6)	
Waist circumference	94.1 ± 14.2	103.1 ± 13.9	93.0 ± 13.8	< .001	106.9 ± 11.7	88.6 ± 11.3	< .001
BMI (categories)				< .001			< .001
< 25 kg/m^2^	447 (38.1)	30 (23.4)	418 (39.9)		12 (4.7)	405 (51.3)	
25–30 kg/m^2^	473 (40.3)	50 (39.1)	423 (40.5)		39 (15.2)	384 (48.7)	
≥ 30 kg/m^2^	253 (21.6)	48 (37.5)	205 (19.6)		205 (80.1)	0 (0.0)	
Daily intake of vegetables, fruits or berries				.256			.485
No	216 (18.4)	19 (14.8)	197 (18.8)		52 (20.3)	145 (18.4)	
Yes	950 (81.0)	109 (85.2)	841 (80.5)		202 (78.9)	639 (81.0)	
Exercise regularity				.044			
Never	26 (2.2)	6 (4.7)	20 (1.9)		8 (3.1)	12 (1.5)	< .001
Less than weekly	115 (9.8)	17 (13.3)	98 (9.4)		41 (16.0)	57 (7.2)	
Once a week	183 (15.6)	21 (16.4)	162 (15.5)		47 (18.4)	115 (14.6)	
2–3 times a week	482 (41.1)	40 (31.2)	442 (42.3)		86 (33.6)	356 (45.1)	
Nearly every day	342 (29.2)	42 (32.8)	300 (28.7)		66 (25.8)	234 (29.7)	
Exercise intensity^c^				.093			< .001
Light	326 (27.8)	44 (34.4)	282 (27.0)		90 (35.2)	192 (24.3)	
Medium	722 (61.6)	66 (51.6)	656 (62.8)		132 (51.6)	524 (66.4)	
Hard	30 (2.6)	4 (3.1)	26 (2.5)		2 (0.8)	24 (3.0)	
Exercise average time				.415			.374
< 15 min	36 (3.1)	6 (4.7)	30 (2.9)		10 (3.9)	20 (2.5)	
15—30 min	229 (19.5)	28 (21.9)	201 (19.2)		51 (19.9)	150 (19.0)	
30 min—1 h	629 (53.6)	64 (50.0)	565 (54.1)		129 (50.4)	436 (55.3)	
> 1 h	194 (16.5)	17 (13.3)	177 (16.9)		36 (14.1)	141 (17.9)	
FINDRISC score	9.5 ± 5.3	17.2 ± 4.3	8.6 ± 4.6	< .001	13.4 ± 3.4	7.1 ± 3.8	< .001

Data are n (%) or mean ± SD. We used Chi-squared tests, Fisher’s exact tests and t-tests

Missing variables n (%): living situation 103 (8.8); work situation 3 (0.3); smoking habits 3 (0.3); waist circumference 16 (1.4); exercise regularity 25 (2.1); exercise intensity 95 (8.1); exercise average time 85 (7.3); daily intake of vegetables, fruits or berries 7 (0.6)

^a^Elevated risk of type 2 diabetes was defined as FINDRISC ≥ 15 and/or BMI ≥ 30 kg/m^2^

^b^“Other” includes leave of absence, home staying (without pay), under education, unemployed and other

^c^Response options: Light = “I take it easy, I don’t get out of breath or break a sweat”; Medium = “I push myself until I’m out of breath and break into a sweat”; Hard = “I practically exhaust myself”

In Table 2, we present the participants’ PAM-13 scores and PAM-13 levels and the differences in scores and levels according to sociodemographic characteristics, clinical characteristics, and health-related behaviours. The sample’s mean PAM-13 score was 69.8 (SD 14.8). Women reported higher patient activation scores than men, including a higher proportion of level 4 activation. Most participants across age groups reported patient activation at level 3. We found no differences in patient activation scores or levels according to age, living situation, educational level, work situation, smoking habits, or BMI (Table 2). However, regarding daily intake of vegetables, fruits or berries and exercise (regularity and average time), participants with higher patient activation scores and levels more frequently reported these favourable health-related behaviours (Table 2).

**Table 2:** Differences in patient activation according to sociodemographic characteristics, clinical characteristics and health-related behaviours

	**PAM-13 scores (0–100)**			**PAM-13 levels**				
				**Level 1**	**Level 2**	**Level 3**	**Level 4**	
	N	Mean ± SD	*p*	n (%)	n (%)	n (%)	n (%)	*p*
Total population	1,173	69.8 ± 14.8	-	67 (5.7)	74 (6.3)	619 (52.8)	413 (35.2)	-
Sex			.002					< .001
Men	544	68.3 ± 15.0		36 (6.6)	47 (8.6)	300 (55.2)	161 (29.6)	
Women	629	71.1 ± 14.4		31 (4.9)	27 (4.3)	319 (50.7)	252 (40.1)	
Age categories			.243					.162
< 45 years	305	68.4 ± 14.1		19 (6.2)	21 (6.9)	173 (56.7)	92 (30.2)	
45–54 years	212	69.9 ± 12.5		8 (3.8)	11 (5.2)	126 (59.4)	67 (31.6)	
55–64 years	271	70.4 ± 16.0		16 (5.9)	20 (7.4)	131 (48.3)	104 (38.4)	
> 64 years	385	70.5 ± 15.4		24 (6.2)	22 (5.7)	189 (49.1)	150 (39.0)	
Living situation			.774					.121
Live with others	841	70.0 ± 14.5		42 (5.0)	50 (5.9)	448 (53.3)	301 (35.8)	
Live alone	229	69.7 ± 16.1		20 (8.7)	16 (7.0)	109 (47.6)	84 (36.7)	
Educational level			.226					.314
Primary/secondary	814	69.5 ± 15.0		53 (6.5)	53 (6.5)	427 (52.5)	281 (34.5)	
University/college	359	70.6 ± 14.3		14 (3.9)	21 (5.9)	192 (53.5)	132 (36.8)	
Work situation^a^			.243					.276
Fulltime work	509	69.9 ± 14.5		27 (5.3)	34 (6.7)	275 (54.0)	173 (34.0)	
Part-time work	138	69.8 ± 12.7		4 (2.9)	8 (5.8)	79 (57.2)	47 (34.1)	
On benefits	106	66.9 ± 15.7		12 (11.3)	6 (5.7)	58 (54.7)	30 (28.3)	
Retired	361	70.7 ± 15.4		20 (5.5)	22 (6.1)	175 (48.5)	144 (39.9)	
Other	56	69.5 ± 15.5		3 (5.4)	3 (5.4)	31 (55.3)	19 (33.9)	
Smoking habits			.243					.256
Never smoked	436	70.7 ± 14.9		23 (5.3)	25 (5.7)	217 (49.8)	171 (39.2)	
Former smoker	556	69.4 ± 15.1		33 (5.9)	39 (7.0)	295 (53.1)	189 (34.0)	
Current smoker	178	68.8 ± 13.3		11 (6.2)	10 (5.6)	106 (59.6)	51 (28.7)	
BMI categories			.236					.077
< 25 kg/m^2^	447	70.7 ± 14.9		23 (5.2)	23 (5.2)	231 (51.6)	170 (38.0)	
25–30 kg/m^2^	473	69.6 ± 15.4		34 (7.2)	32 (6.8)	238 (50.3)	169 (35.7)	
≥ 30 kg/m^2^	253	68.8 ± 13.2		10 (4.0)	19 (7.5)	150 (59.3)	74 (29.2)	
Daily intake of vegetables, fruits or berries			.001					< .001
No	216	66.8 ± 14.4		13 (6.0)	24 (11.1)	126 (58.3)	53 (24.5)	
Yes	950	70.5 ± 14.8		54 (5.7)	50 (5.2)	487 (51.3)	359 (37.8)	
Exercise regularity			< .001					< .001
Never	26	65.0 ± 17.2		3 (11.5)	4 (15.4)	13 (50.0)	6 (23.1)	
Less than weekly	155	66.9 ± 23.9		6 (5.2)	14 (12.2)	67 (58.3)	28 (24.3)	
Once a week	183	66.6 ± 14.5		10 (5.5)	16 (8.7)	113 (61.8)	44 (24.0)	
2–3 times a week	482	69.8 ± 15.1		33 (6.9)	28 (5.8)	242 (50.2)	179 (37.1)	
Nearly every day	342	72.7 ± 14.3		14 (4.1)	11 (3.2)	171 (50.0)	146 (42.7)	
Exercise intensity^b^			.083					.572
Light	326	68.6 ± 14.8		19 (5.8)	25 (7.7)	172 (52.8)	110 (33.7)	
Medium	722	70.7 ± 14.8		40 (5.5)	35 (4.9)	381 (52.8)	266 (36.8)	
Hard	30	71.5 ± 13.7		2 (6.7)	2 (6.7)	13 (43.3	13 (43.3	
Exercise average time			.031					.022
< 15 min	36	65.2 ± 14.7		4 (11.1)	4 (11.1)	20 (55.6)	8 (22.2)	
16—30 min	229	68.2 ± 13.7		12 (5.2)	18 (7.8)	135 (59.0)	64 (28.0)	
30 min—1 h	629	70.6 ± 14.9		40 (6.3)	30 (4.8)	317 (50.4)	242 (38.5)	
> 1 h	194	70.8 ± 15.4		6 (3.1)	11 (5.7)	101 (52.0)	76 (39.2)	

Data are n (%) or mean ± SD. We used Chi-squared tests, Fisher’s exact tests, t-tests and one-way ANOVAs

*Abbreviations*: PAM-13 = 13-item Patient Activation Measure. PAM-13 levels: 1 = Disengaged and overwhelmed; 2 = Becoming aware, but still struggling; 3 = Taking action and gaining control; 4 = Maintaining behaviours and pushing further

^a^“Other” includes leave of absence, home staying (without pay), under education, unemployed and other

^b^Response options: Light = “I take it easy, I don’t get out of breath or break a sweat”; Medium = “I push myself until I’m out of breath and break into a sweat”; Hard = “I practically exhaust myself”

**Fig. 2:**
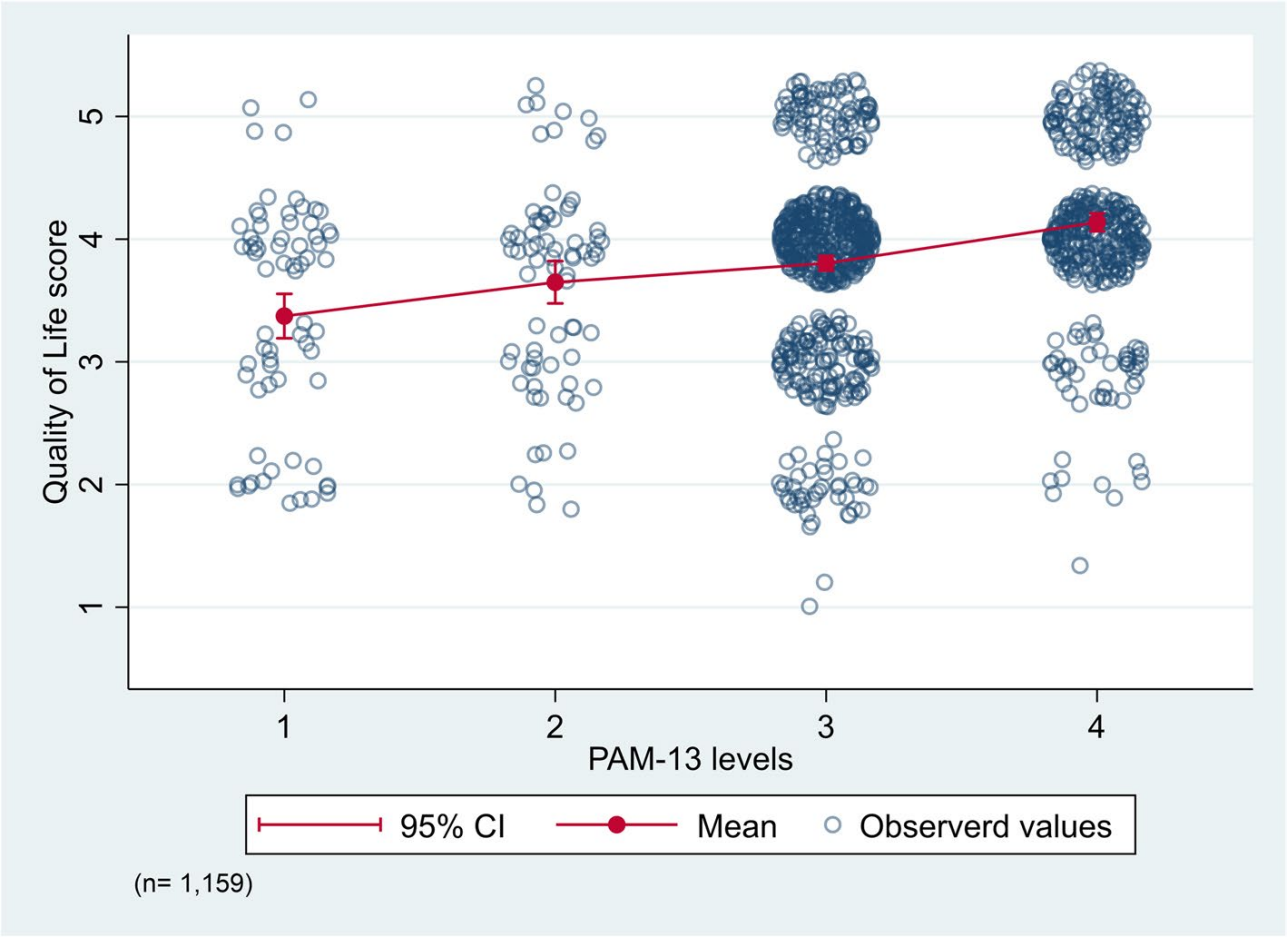
Differences in quality of life score and patient activation levels among adults attending general practice. Observations are jittered by adding random noise before plotting. Abbreviations: PAM-13 = 13-item Patient Activation Measure; 95% CI = 95% Confidence Interval. Quality of Life scores: 1 = very poor, 2 = poor, 3 = neither poor nor good, 4 = good, 5 = very good. PAM-13 levels: 1 = Disengaged and overwhelmed, 2 = Becoming aware, but still struggling, 3 = Taking action and gaining control, 4 = Maintaining behaviours and pushing further

**Fig. 3:**
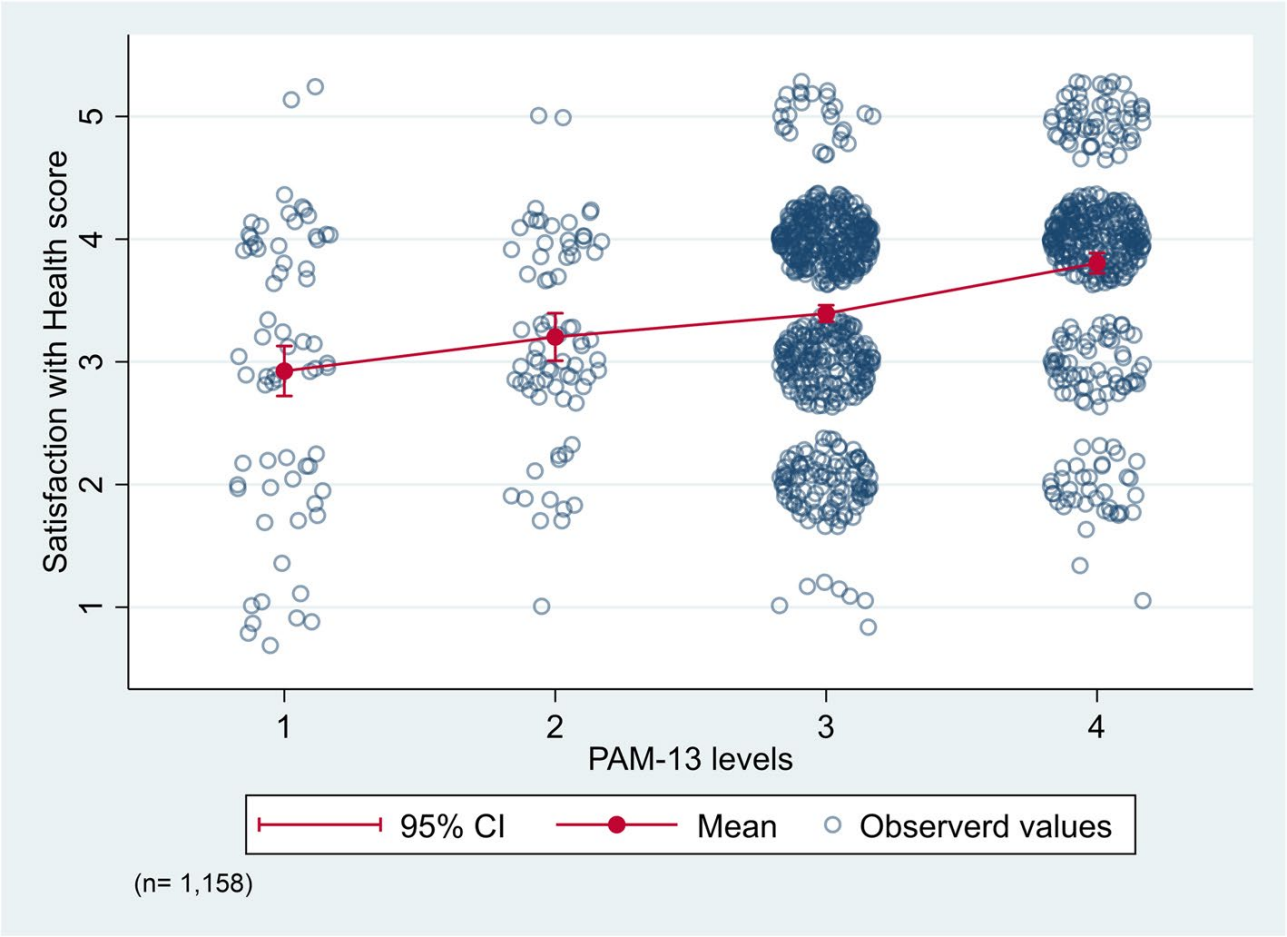
Differences in satisfaction with health score and patient activation levels among adults attending general practice. Observations are jittered by adding random noise before plotting. Abbreviations: PAM-13 = 13-item Patient Activation Measure; 95% CI = 95% Confidence Interval. Satisfaction with Health scores: 1 = very dissatisfied, 2 = dissatisfied, 3 = neither dissatisfied nor satisfied, 4 = satisfied, 5 = very satisfied. PAM-13 levels: 1 = Disengaged and overwhelmed, 2 = Becoming aware, but still struggling, 3 = Taking action and gaining control, 4 = Maintaining behaviours and pushing further

The participants’ quality of life and satisfaction with health (WHOQOL-BREF) scores were respectively 3.9 (SD 0.8) and 3.5 (SD 0.9). We found significant positive correlations between the quality of life score and the PAM-13 score (Spearman’s rho 0.27, *p* < 0.001) and between the satisfaction with health score and the PAM-13 score (Spearman’s rho 0.31, *p* < 0.001). From patient activation levels 1 to 4, the mean quality of life and satisfaction with health scores increased (Fig. 2 & Supplementary table 1 in Additional file 1). Specifically, the quality of life score increased from 3.4 (SD 0.9) in adults reporting patient activation level 1 to 4.1 (SD 0.7) in those reporting level 4. Correspondingly, the satisfaction with health score increased from 2.9 (SD 1.1) to 3.8 (SD 0.8) (Fig. 3 & Supplementary table 1). Further, the most common score combinations were ‘good’ quality of life (score 4) and patient activation level 3 (“Taking action and gaining control”) (Fig. 2) and being ‘satisfied’ with one’s health (score 4) and patient activation level 3 (Fig. 3).

A subset of 128 (10.9%) participants had self-reported diabetes, and 206 (17.6%) scored ≥ 15 on the FINDRISC. We identified that 256 (21.8%) participants had an elevated T2D risk. We present data about their characteristics and health-related behaviours in Table 1. Briefly, participants with T2D or elevated T2D risk were older, had a lower educational level, fewer worked full-time and fewer exercised regularly compared to those without T2D or elevated T2D risk. We found no significant differences in patient activation scores and levels among participants with and without T2D or those with and without elevated T2D risk (Table 3).

**Table 3:** Patient activation in persons with or without diabetes and with or without elevated T2D risk

	**Total study population**	**Self-reported diabetes**			**Elevated T2D risk**^a^		
		**Yes**	**No**		**Yes**	**No**	
	N = 1,173	n = 128	n = 1,045	*p*	n = 256	n = 789	*p*
PAM-13 scores (0–100)	69.8 ± 14.8	70.9 ± 15.9	69.7 ± 14.6	.400	68.5 ± 13.0	70.5 ± 15.1	.110
PAM-13 levels				.502			.089
Level 1—Disengaged and overwhelmed	67 (5.7)	7 (5.5)	60 (5.7)		12 (4.7)	48 (6.1)	
Level 2—Becoming aware, but still struggling	74 (6.3)	10 (7.8)	64 (6.1)		18 (7.0)	46 (5.8)	
Level 3—Taking action and gaining control	619 (52.8)	60 (46.9)	559 (53.5)		152 (59.4)	407 (51.6)	
Level 4—Maintaining behaviours and pushing further	413 (35.2)	51 (39.8)	362 (34.7)		74 (28.9)	288 (36.5)	

Data are mean ± SD or n (%). We used t-tests and Chi-squared tests

*Abbreviations*: *T2D*  type 2 diabetes, *PAM-13*  13-item Patient Activation Measure

^a^Elevated risk of T2D defined as FINDRISC ≥ 15 and/or BMI ≥ 30 kg/m^2^

PAM-13 levels and related scores: level 1 = 0–47.0, level 2 = 47.1–55.1, level 3 = 55.2–67.0, level 4 = 67.1–100

## Discussion

In this study, we found that quality of life and satisfaction with health (WHOQOL-BREF) scores were positively correlated with patient activation scores (PAM-13). Correspondingly, the WHOQOL-BREF scores increased with higher PAM-13 levels. Half of the adults recruited from general practice reported patient activation scores at level 3 (“Taking action and gaining control”), which corresponds with a perception of relatively good confidence and ability to manage one’s health and healthcare. One-third reported the highest level of patient activation (level 4, “Maintaining behaviours and pushing further”). Furthermore, we identified that participants who reported favourable health-related behaviours, such as daily intake of vegetables, fruits or berries and regular exercise, also reported higher PAM-13 scores and levels than participants not reporting these behaviours. We found no differences in patient activation scores or levels among groups of participants with and without T2D or with and without elevated T2D risk.

The participants’ relatively high patient activation scores and levels resemble the results reported in a study comparing PAM-13 data from four European countries [33]. According to Hibbard & Greene [1], people who report high patient activation seem to have higher-quality interactions with their healthcare providers, more positive experiences with care, and fewer problems with coordinating care. From a health promotion and public health perspective, the high scores are positive since patient activation as a concept seems to mediate health outcomes [1, 6]. Highly activated patients may have greater confidence in managing their health and healthcare either because of higher levels of knowledge and abilities or because of better skills to elicit what they need from their healthcare providers. Correspondingly, it is particularly important to identify people with low patient activation.

In our sample, 141 (12.0%) participants reported patient activation levels 1 or 2 (“Disengaged and overwhelmed” or “Becoming aware, but still struggling”). People reporting low patient activation levels tend to have low engagement, knowledge levels and skills to manage their health [1]. Our findings of low activation being associated with less regular physical activity, lower average exercise time and less healthy eating are supported by previous research [1, 11, 34]. Furthermore, people who practice this kind of unfavourable lifestyle have an increased risk of becoming overweight and obese, which in turn increases their risk of developing T2D [14, 15]. Low patient activation is also associated with higher odds of developing T2D [4]. In general, people with T2D need comprehensive follow-up to be able to live well with the condition [35, 36]. When combining T2D and low patient activation, previous research has shown that people report less diabetes knowledge [11], are more likely to need hospitalisation [4–6], have poorer health status and lower educational levels [37] compared to people with high patient activation. Therefore, identifying people with low patient activation will probably be a good investment for the individual, the healthcare services and public health. Here targeted patient activation and self-management interventions that fit individual needs are important [12, 34]. Fortunately, patient activation seems to be a modifiable factor influencing health and disease outcomes. Interventions to increase patient activation among people with T2D have been found to improve important health outcomes such as blood pressure, cholesterol, fat intake, physical activity, smoking status, glucose self-monitoring, glycaemic control, foot care, self-efficacy, diabetes distress, quality of life and symptoms of depression [12, 34, 38].

This study’s findings did not support Sacks et al. [4], who found that people with T2D were more likely to report lower patient activation scores compared to people without diabetes. In that study, activation level 4 was markedly lower in the diabetes group (24.7%) compared to the other groups (31.0–34.5%) [4]. The difference between Sacks et al.’s and our results could be due to many factors, among them the comparatively low number of participants with T2D in our sample, which also seems to self-manage relatively well. Whereas the T2D group in Sacks et al. reported high rates of depression (27.6%) [4], which is known to negatively affect diabetes management [39] and increase the risk of developing diabetes complications [40]. Interestingly, the patient activation levels among patients with T2D reported by Donald et al. [2] strongly resemble our results. However, they did not compare the results to people without diabetes. Further investigation into patient activation among people with T2D is warranted.

Like Magnezi et al. [9], we found a positive correlation between the quality of life and patient activation scores. The correlations between patient activation and, respectively, quality of life and satisfaction with health may appear weak. However, when correlating behavioural or psychosocial variables, the coefficients achieved are typically in the range of 0.30 to 0.40 ([41], p. 377), as found in the present study. However, we also identified that the quality of life and satisfaction with health scores increased across the four patient activation levels. In our analyses, we defined the quality of life and satisfaction with health scores as dependent variables, therefore viewing patient activation as the independent variable possibly affecting quality of life and satisfaction with health. Although the associations may very well be bidirectional, the findings suggest that people with a more positive outlook or perspective on their life and health also have more stamina and are more capable and prepared to take on responsibility and control over their health. Nevertheless, these aspects also need further investigation.

### Implications for care

People with low patient activation are generally less likely to engage in beneficial health-related behaviours compared to people with high patient activation. Therefore, assessing peoples’ skills and knowledge is essential in person-centred follow-up, especially for people with chronic conditions [10]. Patient activation assessments might facilitate a better understanding of risks, such as the relationships between low patient activation, unfavourable health-related behaviours and T2D. Data from the PAM-13 can alert healthcare providers about patients they can expect will benefit from lifestyle-related follow-up and support [4, 6]. Furthermore, healthcare providers who become aware of patients’ need for guidance may be able to approach patients in a more person-centred and successful manner which may further encourage the patients’ engagement in care [42], thereby promoting individualised counselling and support. For people with conditions such as obesity, prediabetes and T2D, it is essential that the healthcare systems and healthcare providers support their ability to keep up with the treatment as active partners in self-managing their health in everyday life [16].

Efforts in general practice to prevent obesity and delay T2D development are good health investments. Unfortunately, the GPs’ heavy workload often results in lower priorities for preventive care [43–46]. Nevertheless, identifying and guiding patients with unfavourable health-related behaviour towards a healthier lifestyle is an important task for general practice [47]. An individualised follow-up to support the patient’s ability to care for their health should ideally be tailored to their patient activation level [1]. For example, less activated people can be encouraged to take small, manageable actions they are likely to succeed at, whereas more activated people may take on more significant behaviour changes [1].

### Strengths and limitations

The study’s main strengths are the large study sample and the high participation rate from four study settings, potentially increasing generalisability. Also, we have used established and validated patient-reported outcome measures. One limitation is that we lack data about the participants’ diversity of diagnoses, comorbidities and/or reasons for seeing their GP. The study’s representativeness is limited to people actively seeking an appointment with their GP. In addition, people seeing their GP are already showing some activation level. Further, the T2D diagnosis was self-reported, but this was later confirmed by checking the patients’ records. According to Midthjell et al. [48], this type of self-report is a reliable source of information in Norwegian health surveys. Moreover, people without considerable health problems may not have found the PAM-13 relevant, possibly contributing to missing data. Finally, our cross-sectional study design prohibits us from inferring whether improved quality of life and satisfaction with health scores lead to increased patient activation scores or vice versa. Despite its limitations, the study should provide a reasonable representation of patient activation scores among Norwegian adults attending general practice.

## Conclusion

By investigating patient activation in a sample of adults in general practice in Norway, we found that 88% of the participants reported activation level 3 or 4. Further, higher patient activation was associated with favourable health-related behaviours, a better quality of life and satisfaction with health. Assessing patient activation has the potential to help GPs identify patients who might benefit from closer follow-up in advance of negative health outcomes.

## Supplementary information

**Additional file 1: Supplementary Table 1.** Quality of life and satisfaction with health scores by patient activation levels among adult patients.

## Data Availability

The dataset used for the current study is available from the corresponding author on reasonable request.

## Abbreviations

BMI: Body Mass Index
FINDRISC: Finnish Diabetes Risk Score
GP: General Practitioner
PAM-13: Patient Activation Measure (13 items)
RCT: Randomised Controlled Trial
T2D: Type 2 Diabetes
WHOQOL-BREF: World Health Organisation Quality of Life-BREF questionnaire
