# Supplementary material for: Patient activation in adults attending appointments in general practice: a cross-sectional study
Source: BMC Prim Care. 2023 Jul 10;24:144. doi: 10.1186/s12875-023-02102-9 (PMC10331983; doi:10.1186/s12875-023-02102-9)
Supplement: Supplementary file 1 — Additional file 1: Supplementary Table 1. Quality of life and satisfaction with health scores by patient activation levels among adult patients. [file 12875_2023_2102_MOESM1_ESM.pdf]

Patient activation in adults attending scheduled appointments in general practice: a cross-sectional study.

Hernar, Graue, Igland, Richards, Riise, Haugstvedt & Kolltveit (2023) BMC Primary Care. https://doi.org/10.1186/s12875-023-02102-9

Supplementary table 1. Quality of life and satisfaction with health scores by patient activation levels among adult patients.

	**Quality of Life score***			**Satisfaction with Health score†**		
	N= 1,159	Mean ± SD	*p*	N= 1,158	Mean ± SD	*p*
Patient Activation Measure (PAM-13) levels			<.001			<.001
Level 1 - Disengaged and overwhelmed	n= 67	3.4 ± .9		n= 67	2.9 ± 1.1	
Level 2 - Becoming aware, but still struggling	n= 74	3.6 ± .8		n= 74	3.2 ± .8	
Level 3 - Taking action and gaining control	n= 611	3.8 ± .8		n= 609	3.4 ± .9	
Level 4 - Maintaining behaviours and pushing further	n= 407	4.1 ± .7		n= 408	3.8 ± .8	

***** Scored from 1-5; 1 = very poor, 2 = poor, 3 = neither poor nor good, 4 = good, 5 = very good.
† Scored from 1-5; 1 = very dissatisfied, 2 = dissatisfied, 3 = neither dissatisfied nor satisfied, 4 = satisfied, 5 = very satisfied.
